# Anatomical Approaches for Insertion Sites of External Ventricular Drainage Catheters

**DOI:** 10.7759/cureus.109187

**Published:** 2026-05-19

**Authors:** Menatalla M ElBadway, Muhammad Mohsin Khan, Salwa Al-Maraghi, Ali Msheik, Jawad Yousaf, Ghaya Al-Rumaihi

**Affiliations:** 1 Clinical Sciences, College of Medicine, Qatar University - QU Health, Doha, QAT; 2 Clinical Research, University of Dresden, Dresden, DEU; 3 Neurological Surgery, Hamad Medical Corporation, Doha, QAT

**Keywords:** acute hydrocephalus, catheter placement, cerebrospinal fluid (csf), external ventricular drainage, external ventricular drain (evd), intracranial pressure, neurosurgical anatomy, ventriculostomy guide

## Abstract

External ventricular drains (EVDs) are widely used in neurosurgery to control intracranial pressure, divert cerebrospinal fluid (CSF), and facilitate monitoring in acute neurological and neurosurgical conditions. Accurate catheter placement is essential, as anatomical trajectory and insertion site directly influence efficacy and complication rates. This review summarizes the major anatomical entry points for EVD insertion, including Kocher’s, Frazier’s, Keen’s, occipital-parietal, and Dandy’s points, and discusses the rationale, advantages, and limitations associated with each approach. Commonly used techniques, such as freehand landmark-based placement, stereotactic guidance, neuronavigation, ultrasound-assisted insertion, and endoscopic methods, are reviewed in the context of accuracy and clinical applicability. Reported outcomes demonstrate high cannulation success with standard approaches, while complications such as infection, hemorrhage, catheter misplacement, and obstruction remain significant considerations. Key patient-, procedure-, and maintenance-related risk factors are highlighted, along with evidence-based recommendations to improve safety and reduce variability in clinical practice. As technological developments continue to advance catheter design and placement guidance, understanding anatomical strategies and standardized care protocols remains essential for optimizing EVD effectiveness. This review provides a comprehensive, practical overview intended to support neurosurgeons and critical care clinicians in selecting safe insertion sites and minimizing procedure-related complications.

## Introduction and background

Since the early development of the field of neurosurgery, various techniques and methods have been attempted and used to drain the cranium, ranging from simple metal rods to various types of catheters. These early efforts showed promising results initially but frequently resulted in devastating mortalities.

As the tools developed, safer and more biocompatible instruments were adopted for intracranial use. The addition of pumps and probes to measure intracranial pressure and regulate the volume of fluid drained substantially improved patient outcomes and established external ventricular drains (EVDs) as fundamental equipment in neurosurgery, intensive care, and trauma management.

As part of the evolution of EVDs and with accumulating knowledge about the brain and the ventricular system, it became increasingly clear that the anatomical precision of catheter insertion has a profound impact on neurosurgical patient outcomes. Placement is typically determined by patient pathology, the specific component of the ventricular system targeted for drainage, and the optimal entry point from a patient safety standpoint. In this paper, we discuss the different anatomical access points, placement techniques, outcomes, and efficacy.

## Review

Indication for EVD

In concept, EVDs are used to drain excess cerebrospinal fluid (CSF), arising from excess secretion, depressed absorption, or obstruction, impeding normal ventricular flow [1,2]. Since the cranium constitutes a closed, rigid compartment, any of these factors will directly affect pressure, flow, and perfusion to the brain parenchyma, producing symptoms such as headache, visual disturbances, or other compressive features depending on the region of the brain involved.

Another indication is rapid intracranial accumulation, whether from CSF, blood, or edema, rendering EVD insertion either emergent or elective [3]. EVDs are indicated for elevated intracranial pressure (ICP) due to obstructive or communicating hydrocephalus, intraventricular hemorrhage (IVH) from chronic or acute trauma, traumatic brain injury (TBI) with increased ICP, or mass effect from edema, abscess, or tumor.

In conditions where the CSF itself is infected or hemorrhagic, drainage becomes paramount. In cases of primary ventriculitis/meningitis or secondary infections following neurosurgical interventions, EVD is the method of choice [4,5]. Similarly, EVD is indicated for IVH resulting from trauma.

EVDs are routinely employed in TBI for partial or multimodal monitoring of ICP, particularly in severe cases admitted to the intensive care unit (ICU) [6,7]. In patients with tumors or masses compressing the ventricular system who are awaiting definitive treatment, EVD placement serves as a bridge to ventriculoperitoneal (VP) shunt insertion, tumor resection, or third ventriculostomy.

Another well-established indication is the postoperative setting following major neurosurgical procedures in which edema, hemorrhage, or obstruction of CSF pathways is anticipated [3]. For example, after resection of large posterior fossa tumors, particularly those involving the fourth ventricle or brainstem, there is a significant risk of postoperative hydrocephalus due to blockage of CSF flow or impaired absorption. In such cases, a temporary EVD provides CSF diversion, helps control ICP, prevents acute deterioration, and allows assessment of CSF characteristics, while also serving as a window to evaluate the need for permanent shunting if hydrocephalus persists postoperatively.

Placement techniques

EVDs can be inserted at the bedside or in an operating room (OR) setting. Regardless of location, the procedure must adhere to strict aseptic technique and be performed as a sterile procedure to prevent infection and contamination [4,5].

The choice of placement technique depends on the patient’s anatomy and clinical context. The freehand technique remains the most commonly used approach because of its simplicity and practicality. However, image-guided placement using neuronavigation is preferred in patients with distorted ventricular anatomy or prior cranial surgery, where greater placement accuracy is required [8,9].

A more precise, though time-consuming, method is stereotactic-guided placement. This technique is typically reserved for elective EVD insertions in cases involving small or deep ventricular systems [10,11]. Due to its setup complexity and time requirements, it is less commonly used in emergencies.

In neonates, ultrasound-guided placement is preferred given the anatomical accessibility afforded by open fontanelles. It is also commonly used intraoperatively, offering real-time guidance through an open craniotomy or via a burr hole [12].

Patients undergoing endoscopic procedures may receive endoscopic-assisted EVD placement, which allows direct visualization of the catheter entry point and improves targeting accuracy [13].

Despite the availability of multiple techniques, the standard freehand method remains most widely used. It relies heavily on anatomical landmarks and the surgeon's experience [8,14]. The procedure typically involves creating a burr hole at the access point, followed by dural opening and advancing the catheter toward the ipsilateral foramen of Monro to a depth of 5-7 cm in adults. Following insertion, the catheter is commonly tunneled subcutaneously for several centimeters away from the burr hole exit site before externalization. Adequate tunneling and secure fixation help reduce CSF leakage, infection risk, and accidental catheter dislodgement, particularly in agitated or frequently repositioned patients [4]. When inserted intraoperatively, standard neurosurgical instruments are used alongside preoperative CT or MRI images uploaded to a neuronavigation system for real-time guidance [9]. Currently, several companies provide neuronavigation devices with reduced setup times and user-friendly interfaces (e.g., Brainlab, StealthStation, and Medtronic).

Stereotactic-guided placement is particularly useful in patients with previously failed freehand attempts or those with small, hard-to-access ventricles. It offers high-precision targeting using a fixed head frame and a coordinate system [10,11]. However, due to the longer setup time, its use in emergency settings remains limited.

Insertion sites and anatomical approaches

The primary goal when inserting an EVD is to access the ventricular system. This can be achieved through several target points, such as the frontal horn (anterior to the foramen of Monro), the occipital horn, or the temporal horn of the lateral ventricles. Specific anatomical landmarks are employed in clinical practice to guide the catheter safely and effectively [3,8,15]. *Kocher's Point (Frontal Approach)*

**Figure 1:**
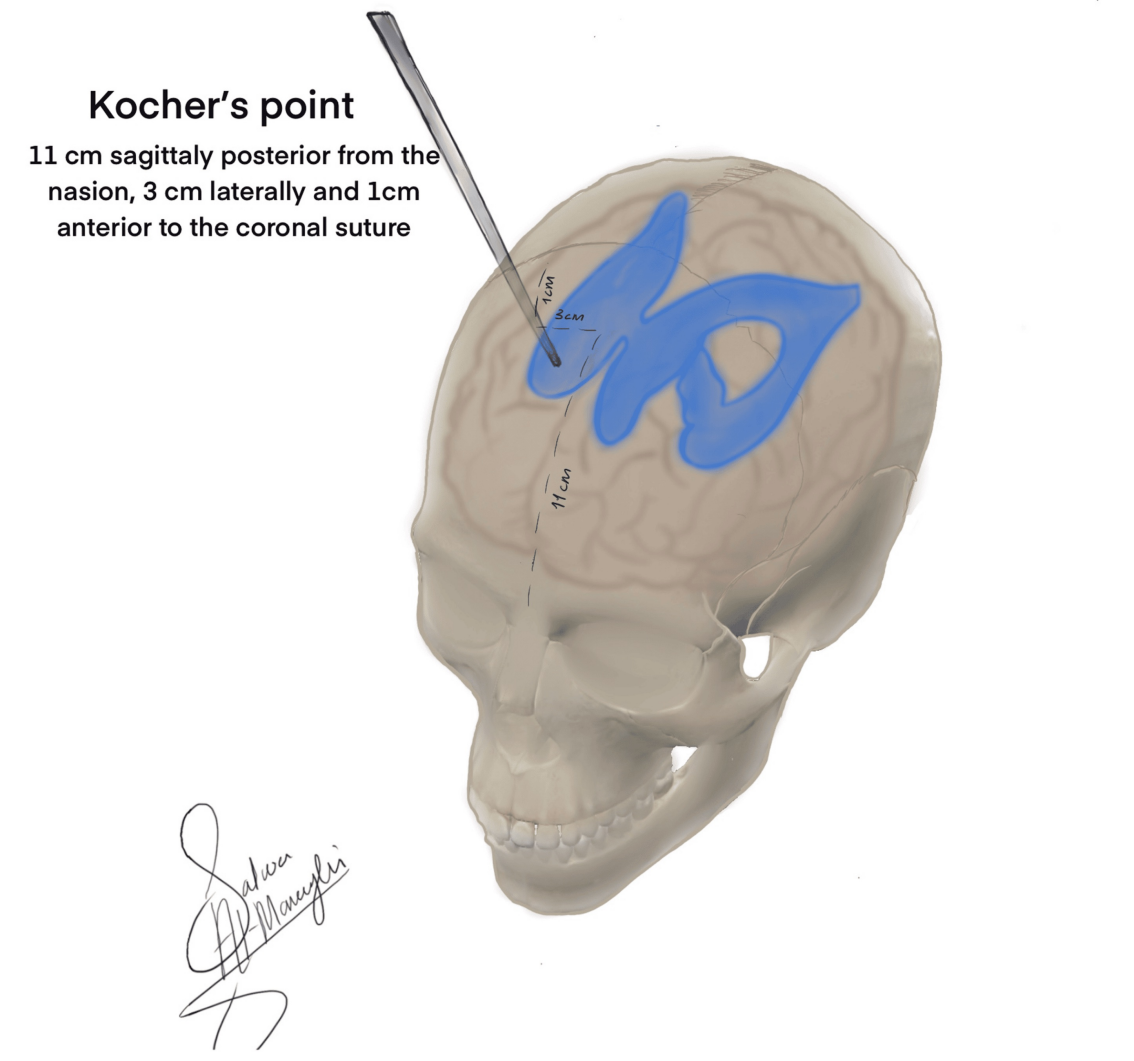
Kocher's EVD insertion point. Illustration demonstrating commonly used cranial entry sites for external ventricular drain (EVD) placement. Kocher’s point is positioned approximately 11 cm posterior to the nasion along the sagittal plane, 3 cm lateral to the midline, and 1 cm anterior to the coronal suture. These landmarks guide safe catheter insertion into the lateral ventricles. The figure is an original hand-drawn illustration created by Dr. Salwa Al-Maraghi using Sketchpad (Sketch.IO, Portland, Oregon).

Kocher's point depicted in Figure 1 is the most commonly used and generally considered the safest access site [8]. It is located 11 cm posterior to the nasion, 3 cm lateral to the midline, and 1 cm anterior to the coronal suture. This trajectory accesses the frontal horn and terminates near the anterior aspect of the foramen of Monro. It is preferred because it avoids eloquent cortex and typically yields reliable drainage outcomes.

Frazier's Point (Parietal Approach)

**Figure 2:**
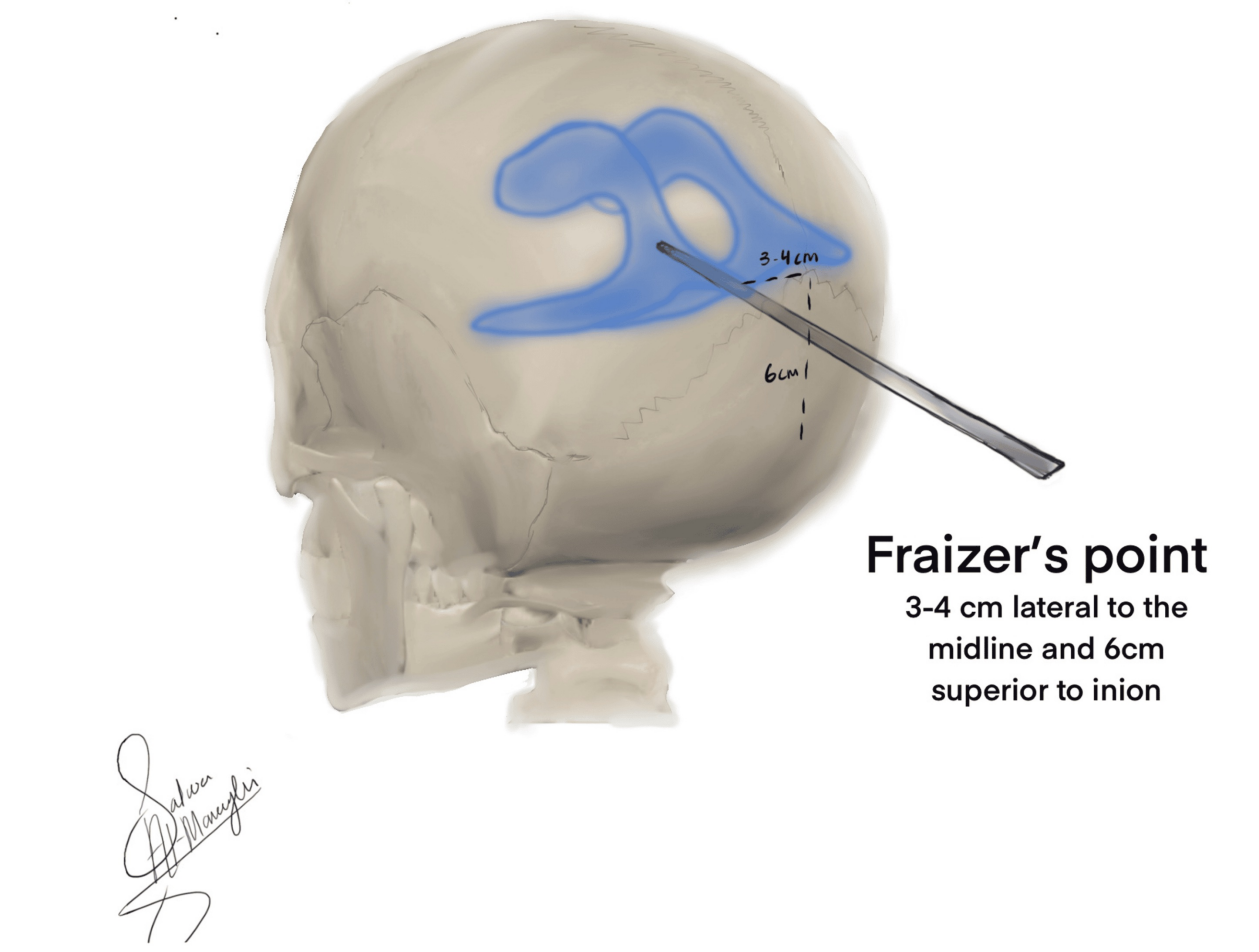
Fraizer's EVD insertion point. Illustration demonstrating commonly used cranial entry sites for external ventricular drain (EVD) placement. Frazier’s point lies 3-4 cm lateral to the midline and 6 cm superior to the inion. These landmarks guide safe catheter insertion into the lateral ventricles. The figure is an original hand-drawn illustration created by Dr. Salwa Al-Maraghi using Sketchpad (Sketch.IO, Portland, Oregon).

Frazier's point depicted in Figure 2 is located approximately 3-4 cm off the midline and 6 cm above the inion, providing access to the occipital horn and targeting the trigone of the lateral ventricle [3]. It is used when the frontal route is contraindicated, for example, in the setting of a frontal tumor or trauma. The proximity to venous sinuses and cortical veins increases the hemorrhagic risk of this approach.

Keen's Point (Temporal Approach)

**Figure 3:**
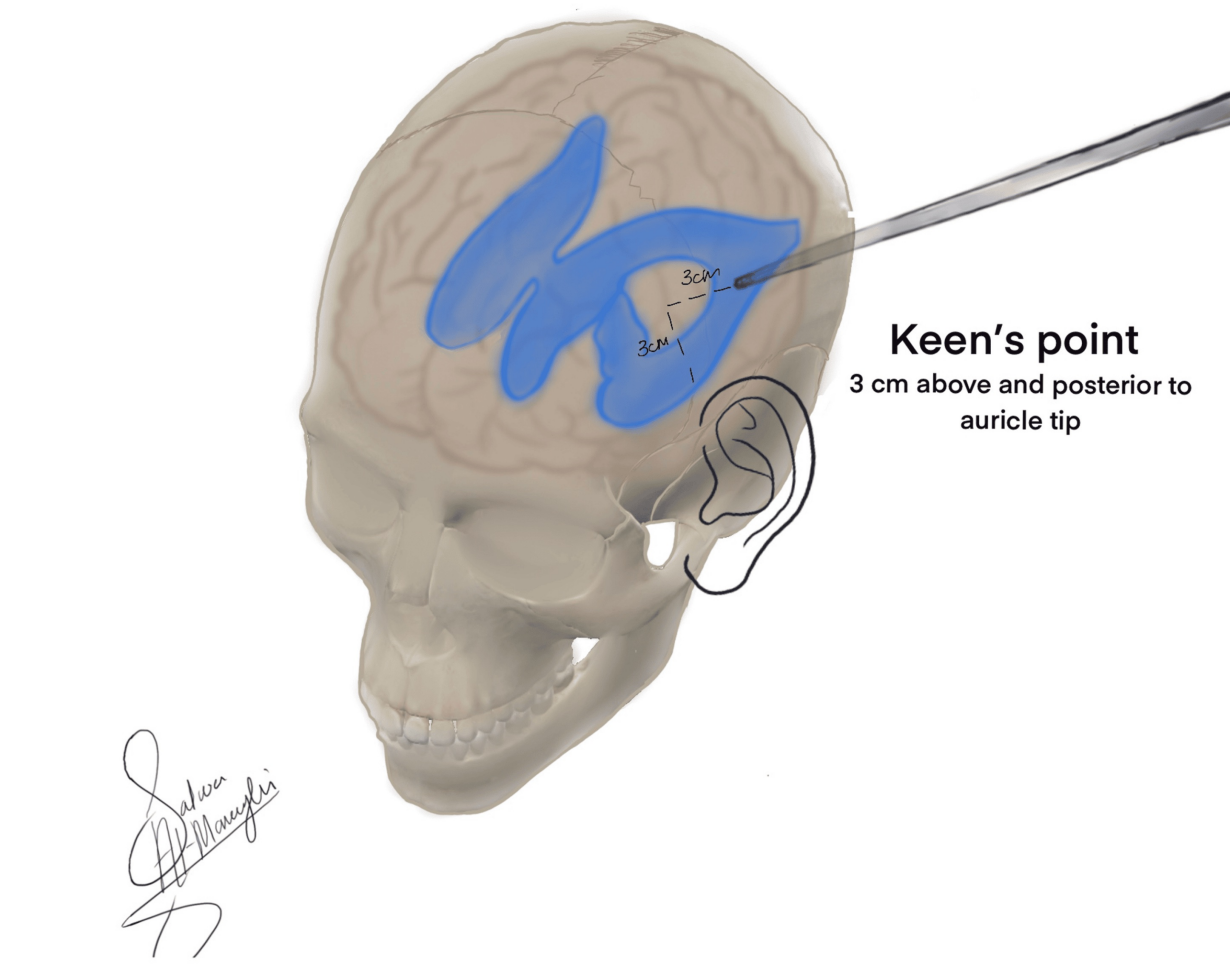
Keen's EVD insertion point. Illustration demonstrating commonly used cranial entry sites for external ventricular drain (EVD) placement. Keen’s point is located approximately 3 cm above and posterior to the auricular tip. These landmarks guide safe catheter insertion into the lateral ventricles. The figure is an original hand-drawn illustration created by Dr. Salwa Al-Maraghi using Sketchpad (Sketch.IO, Portland, Oregon).

Keen's point depicted in Figure 3 is situated approximately 2.5-3 cm superior and posterior to the pinna of the ear, allowing access through the temporal horn [3]. It is reserved for cases in which the frontal approach is not feasible and carries a greater risk due to proximity to deep vessels and functionally eloquent regions.

Occipital-Parietal Approach

The occipital-parietal point is defined radiologically as the trajectory with the shortest parenchymal distance from the atrium of the lateral ventricle to the skull surface [3]. Anatomically, it can be approximated as the midpoint between Keen's and Frazier's points within a few millimeters, with the trajectory directed toward the midline at 4 cm above the nasion.

Dandy's Point (Posterior Parietal Approach)

Dandy's point is located 2 cm off the midline and 3 cm above the inion, accessing the body of the lateral ventricle [3]. This route is now infrequently used due to poor operative visualization and unfavorable angles.

Alternative Approaches

In cases where none of the standard access points are safe or anatomically feasible, such as patients with anomalous anatomy or prior cranial surgery, a custom trajectory can be planned using CT or MRI guidance to identify a patient-specific safe corridor into the ventricular system [10,11].

Outcomes and efficacy

Freehand placement of EVDs via Kocher's point achieves successful ventricular cannulation in 85-90% of cases, with rates exceeding 95% when paired with image guidance or stereotactic assistance [8,9].

EVDs are not curative and serve as a temporary measure to preserve cerebral perfusion pressure and enable CSF sampling and ICP monitoring [1,2]. Favorable neurological outcomes (Glasgow Outcome Scale > 3) are linked to timely placement, particularly in TBI, aneurysmal subarachnoid hemorrhage (SAH), and obstructive hydrocephalus [6,7].

EVDs are highly effective for immediate reduction of elevated ICP through CSF drainage and enable accurate maintenance of ICP within desired targets (<20 mmHg) through controlled drainage and continuous or intermittent monitoring [2,7]. In TBI and SAH, EVDs are often employed as first-line tools in tiered ICP management protocols, particularly when combined with sedation, hyperosmolar therapy, or surgical decompression.

EVD placement does not directly improve mortality; however, it prevents irreversible injury and herniation by enabling detection and management of acute pressure increases, and reduces hydrocephalus-related neurological deterioration [6,16]. Prolonged EVD use increases the risk of ventriculostomy-associated infection, which can adversely influence outcomes if not appropriately monitored [4,5]. Although there is no clear consensus guideline on the frequency of EVD replacement, this remains an area of active clinical judgment.

Complications and troubleshooting

Although EVDs are indispensable in modern neurosurgical and critical care practice, they carry notable risks. Complications may arise during insertion, throughout the course of drainage, or at the time of catheter removal. Understanding these complications and the principles of troubleshooting is critical for maintaining patient safety and improving outcomes [3,13,16].

Catheter Misplacement

Catheter misplacement is one of the most frequent complications, particularly with freehand techniques. Misplacement may involve lodging the catheter in brain parenchyma, subarachnoid space, or contralateral ventricle, leading to insufficient drainage or erroneous ICP readings [14,17]. Misplacement rates range from 10% to 40% depending on operator experience and ventricular size [17,18]. Troubleshooting begins with confirmatory CT imaging when drainage is inadequate or ICP values are inconsistent with the clinical examination. Catheters with suboptimal positioning that remain functional may be left in situ; non-functional misplacements typically require revision under image guidance [9,18].

Hemorrhage

EVD insertion carries a risk of tract hemorrhage, typically small and clinically silent. Symptomatic hemorrhage is uncommon (<2%), but its incidence increases with multiple insertion passes, coagulopathy, and distorted anatomy [19]. If bleeding is identified post insertion, management depends on size and symptomatology. Asymptomatic hemorrhage is monitored with serial imaging; clinically significant hemorrhage requires reversal of coagulopathy, ICP control, and, in rare cases, surgical evacuation [15,19].

Infection

Ventriculostomy-associated infections (VAI), including ventriculitis and meningitis, represent the most serious long-term complication of EVD use. Infection rates vary widely (2-27%) and increase with prolonged catheter duration, frequent CSF sampling, and breaches of sterility [4,5,20]. Management requires a high index of clinical suspicion, particularly in the presence of fever, rising ICP, worsening neurological status, or turbid CSF. Diagnostic CSF sampling is performed using a strict aseptic technique. Treatment includes systemic antibiotics, catheter removal or exchange, and intraventricular antibiotics in severe, multiloculated infections.

Catheter Obstruction or Malfunction

Catheter blockage is common in patients with IVH or purulent ventriculitis, arising from clot formation, debris, or catheter kinking [21]. Troubleshooting includes re-zeroing and flushing the drainage system (never intraluminally unless per protocol), repositioning the patient to facilitate flow, and gentle irrigation under strict aseptic conditions in selected cases. Persistent obstruction typically necessitates catheter replacement.

CSF Leakage and Dislodgement

CSF leakage around the exit site increases infection risk and may reflect poor tunneling or inadequate catheter fixation [21]. Catheter dislodgement occurs more frequently in agitated or pediatric patients [12]. Troubleshooting includes reinforcing securement devices, retaping the catheter, or replacement if the catheter integrity is compromised. Persistent CSF leaks require system revision and reassessment of the tunneling technique. Catheter fixation typically involves suturing and secure dressing techniques to minimize accidental displacement during patient movement or nursing care.

Overdrainage and Underdrainage

Improper leveling of the drainage system may cause overdrainage, risking slit ventricles, subdural collections, or herniation, or underdrainage, resulting in ICP elevation [22]. Troubleshooting centers on verifying that the transducer is leveled to the tragus (approximating the foramen of Monro) and confirming drainage height settings. Regular reassessment is essential in patients requiring frequent repositioning.

Risk factors

EVDs are lifesaving but carry patient-related, procedure-related, and maintenance-related risks that must be carefully considered and mitigated [3,13].

Patient-Related Risk Factors

Age is a critical risk factor. Elderly patients face a higher risk of infection and hemorrhage due to physiological fragility. Studies have shown that each additional year of age increases the risk of EVD infection by approximately 4-5.1% [5]. Comorbidities such as diabetes, malignancy, and immunosuppression elevate infection risk and worsen prognosis in EVD-dependent patients. Immunosuppressed patients have been found to carry a significantly higher risk of EVD-related infections [4,5].

Liver disease and coagulopathy increase the risk of hemorrhagic complications. Patients with coagulopathy, such as those with liver cirrhosis, have been reported to have an increased risk of spontaneous hemorrhage following EVD placement [15,19]. Prior cranial surgery is a recognized risk factor, as scar tissue and altered anatomy may complicate catheter insertion. Patients with prior brain surgery involving CSF diversion have a reported hazard ratio of 8.08 for developing EVD-related infections [5].

Anatomical variants, including slit ventricles, midline shifts, or congenital anomalies, increase the risk of catheter misplacement and procedural complications [17].

Procedure-Related Risk Factors

Operator inexperience is associated with higher rates of hemorrhage, misplacement, and infection. Procedures performed by less experienced surgeons are consistently associated with higher complication rates [14]. Freehand techniques carry higher misplacement risks relative to image-guided methods. The use of image guidance has been shown to improve placement accuracy and reduce misplacement rates [18].

Multiple insertion attempts increase the risk of parenchymal injury and infection. One study reported a 14% rate of tract hemorrhage associated with multiple passes [19]. Breaches of sterility and inadequate aseptic technique significantly increase infection risk. Strict adherence to aseptic protocols is essential to minimize EVD-related infections [4].

Short tunneling or inadequate catheter fixation increases the risk of dislodgement and CSF leak [21]. Inaccurate identification of the anatomical access point increases the risk of cortical and vascular injury [15].

Maintenance-Related Risk Factors

Frequent CSF sampling and catheter manipulation are associated with elevated infection risk. Patients whose EVDs were sampled more frequently have been shown to have a significantly higher infection incidence [20]. Prolonged catheter use raises the infection rate significantly, though no consensus exists on optimal catheter duration. Maintaining a single catheter for more than seven days has been associated with a significantly increased risk of EVD-related infections [23].

Unnecessary circuit breaks increase contamination opportunities, and inadequate exit-site care raises the risk of local infection progressing to ventriculitis [21]. Incorrect transducer leveling may produce erroneous ICP readings, leading to inappropriate clinical management and over- or under-drainage [22].

Best practices and current guidelines

Modern neurosurgical and critical care literature increasingly emphasizes standardized protocols for EVD placement, care, and monitoring to reduce complications and improve outcomes. Recommendations are largely derived from neurosurgical society consensus statements, infection-control guidelines, and prospective cohort studies [3,4].

Sterile Technique and Insertion Protocols

EVD insertion must be performed as a fully sterile procedure using maximal barrier precautions, including cap, gown, sterile gloves, large drape, and chlorhexidine skin preparation, similar to central line placement standards [4]. Image guidance is recommended when anatomy is distorted, ventricles are small, or prior freehand attempts have failed [9,18]. Single-pass insertion should be prioritized to minimize tract hemorrhage and infection risk [19].

Post-insertion Care and Monitoring

The EVD system must remain a closed circuit at all times. Unnecessary disconnections should be avoided, and all sampling ports must be appropriately decontaminated before access [21]. ICP transducers must be leveled to the tragus, with reassessment whenever the patient is repositioned [22]. Nursing teams should follow standardized EVD care bundles, including daily site inspections, sterile dressing changes, monitoring of CSF characteristics, and documentation of drainage volumes [21].

CSF Sampling and Infection Prevention

CSF sampling should be limited to clinically indicated situations; routine daily sampling is no longer recommended due to infection risk [20]. Prophylactic systemic antibiotics may be administered peri-procedurally; however, prolonged prophylaxis has not demonstrated benefit and contributes to antimicrobial resistance [4]. Antimicrobial-impregnated catheters (AICs) have been shown to reduce infection rates in high-risk patients and are recommended when available [4,5].

Duration of Use and Replacement Strategies

There is no universal consensus on maximum catheter duration, but infection risk increases significantly after seven days without strict care protocols [23]. Routine scheduled catheter changes are not supported by evidence; replacement is indicated only for infection, obstruction, or malfunction [21]. Conversion to a permanent CSF diversion strategy (e.g., VP shunt) is considered when hydrocephalus persists or prolonged drainage is anticipated.

Future Directions

Advances under active investigation include real-time ultrasound-assisted freehand placement, miniaturized pressure sensors and wireless monitoring devices, robotic stereotactic systems for high-precision catheter insertion, and standardized EVD bundles integrating AI-driven ICP pattern analysis [1,3]. These innovations aim to reduce complications, improve accuracy, and optimize neurocritical care workflows.

## Conclusions

EVDs remain one of the most critical tools in neurosurgery and neurocritical care, providing rapid CSF diversion, ICP control, and diagnostic access across a range of emergent and elective conditions. Their safety and efficacy rely on an intricate balance between anatomical precision, sterile technique, and vigilant postoperative care. While traditional freehand methods remain widely used and effective, modern image-guided and stereotactic techniques have improved placement accuracy, particularly in patients with altered anatomy.

Complications such as infection, hemorrhage, misplacement, and catheter dysfunction, although common, are largely preventable through strict adherence to evidence-based protocols and standardized monitoring practices. Understanding patient-specific, procedural, and maintenance-related risk factors enables clinicians to anticipate challenges and intervene early. As technology continues to advance, future innovations in navigation, catheter materials, and neuro-monitoring hold promise for reducing complications and enhancing patient outcomes. EVDs will remain a cornerstone of acute neurosurgical care, and ongoing refinement of practice guidelines will continue to shape safer and more effective approaches to their use.
